# Reoccurring *Escherichia coli* O157:H7 Strain Linked to Leafy Greens–Associated Outbreaks, 2016–2019

**DOI:** 10.3201/eid2909.230069

**Published:** 2023-09

**Authors:** Jessica C. Chen, Kane Patel, Peyton A. Smith, Eshaw Vidyaprakash, Caroline Snyder, Kaitlin A. Tagg, Hattie E. Webb, Morgan N. Schroeder, Lee S. Katz, Lori A. Rowe, Dakota Howard, Taylor Griswold, Rebecca L. Lindsey, Heather A. Carleton

**Affiliations:** Centers for Disease Control and Prevention, Atlanta, Georgia, USA (J.C. Chen, K. Patel, P.A. Smith, E. Vidyaprakash, C. Snyder, K.A. Tagg, H.E. Webb, M.N. Schroeder, L.S. Katz, L.A. Rowe, D. Howard, T. Griswold, R.L. Lindsey, H.A. Carleton);; Oak Ridge Institute for Science and Education, Oak Ridge, Tennessee, USA (K. Patel, C. Snyder)

**Keywords:** *Escherichia coli* O157:H7, foodborne disease, sequence analysis, bacteria, enteric infections, United States

## Abstract

Genomic characterization of an *Escherichia coli* O157:H7 strain linked to leafy greens–associated outbreaks dates its emergence to late 2015. One clade has notable accessory genomic content and a previously described mutation putatively associated with increased arsenic tolerance. This strain is a reoccurring, emerging, or persistent strain causing illness over an extended period.

*Escherichia coli* O157:H7 is estimated to cause ≈63,000 domestically acquired foodborne illnesses and 20 deaths in the United States each year (*1*). *E. coli* O157:H7 infections are typically associated with abdominal cramps, bloody diarrhea, and vomiting; however, a rare but serious condition called hemolytic uremic syndrome can develop, resulting in anemia and acute renal failure (*2*). Healthy cattle serve as the main reservoir for *E. coli* O157:H7, and contaminated food, water, and environmental sources, as well as contact with animals, have been the source of outbreaks of *E. coli* O157:H7 infections (*3*,*4*). More recently, contaminated leafy greens have been recognized as a major source of *E. coli* O157:H7 illnesses and outbreaks. In foodborne illness attribution estimates for 2020 based on outbreak data, 58.1% of *E. coli* O157:H7 illnesses were attributed to vegetable row crops, a category that includes leafy greens (https://www.cdc.gov/foodsafety/ifsac/annual-reports.html). During 2009–2018, a total of 32 confirmed or suspected outbreaks of *E. coli* O157:H7 infections linked to contaminated leafy greens occurred in the United States and Canada (*5*).

A large *E. coli* outbreak in late 2019, hereafter referred to as outbreak A, caused 167 cases, hospitalized 85 persons from 27 states, and was associated with the consumption of romaine lettuce from Salinas Valley, California, USA (https://www.cdc.gov/ecoli/2019/o157h7-11-19/index.html). We characterized isolates from outbreak A and highly related isolates by using a variety of molecular methods.

## The Study

A query of the PulseNet database revealed 356 isolates related to the outbreak strain that had <15 core-genome multilocus sequence typing (MLST; cgMLST) allele differences (Table 1; Appendix 1 Table 1) (*6*). Of those, 302 isolates corresponded to human cases associated with 6 outbreaks spanning 3 years; dates of isolation ranged from September 27, 2016, to January 3, 2020. An additional 54 isolates were either clinical isolates not associated with a recognized outbreak (n = 14) or from environmental (n = 20), food (n = 8), or animal (n = 12) samples. Seven-gene MLST and Manning clade typing revealed all isolates were sequence type (ST) 11 and belonged to Manning clade 2 (Appendix 2). In silico PCR of the Shiga toxin (stx) genes revealed that all but 2 isolates contained *stx2a*, whereas 2 remaining isolates had no detectable stx genes. We generated a closed-reference genome, 2019C-3201 (Strain: PNUSAE020169; BioSample: SAMN10432148), using PacBio Sequel technology (https://www.pacb.com) and assembled with Flye version 2.6 (*7*). The sequence data assembled into a single complete chromosomal contig and 3 plasmids (Table 2).

**Table 1:** Summary of outbreaks caused by reoccurring *Escherichia coli* O157:H7 strain REPEXH02 linked to leafy greens–associated outbreaks, 2016–2019*

Outbreak	No. sequences	Timeframe	No. sequences, subset	Median subset allele differences (min–max)	Median hqSNP subset differences (min–max)	Outbreak source	Growing region
D	20	Sep 27–Dec 5, 2016	20	2 (0–5)	3 (0–17)	Unknown	NA
C	23	Nov 10–-Dec 14, 2017	23	0 (0–3)	1 (0–5)	Leafy greens	Likely SW USA, Mexico
B3	7	Jul 31–Aug 15, 2018	7	0 (0–3)	1 (0–10)	Unknown	NA
B2	71	Oct 8–Dec 7, 2018	69	1 (0–4)	2 (0–10)	Romaine lettuce	Santa Maria, CA
B1	19	Nov 1–Dec 18, 2018	18	2 (0–5)	4 (0–10)	Leafy greens	NA
A	179	Sep 27, 2019–Jan 3, 2020	84	1 (0–5)	2 (0–12)	Romaine lettuce	Salinas Valley, CA
Nonhuman samples collected in Santa Maria	23	Nov 14, 2019	12	0 (0–1)	1 (0–5)	NA	Santa Maria, CA
Not associated with known outbreak	14	Oct 18, 2016–Aug 4, 2019	12	1 (0–3)	8 (0–18)	NA	NA
All	356	Sep 27, 2016–Jan 3, 2020	245	2 (0–8)	10 (0–39)	NA	NA

*hqSNP, high-quality single nucleotide polymorphism; NA, not applicable.

**Table 2:** Genomic attributes of the 2019C-3201 reference genome of reoccurring *Escherichia coli* O157:H7 strain REPEXH02 linked to leafy greens–associated outbreaks, 2016–2019*

Contig name	GenBank accession no.	Length, bp	Sequence coverage	Replicon	PTU (*8*)
2019C-3201 chromosome	CP090856	5,488,442	866	None	NA
p2019C-3201_1	CP090857	87,920	732	IncI1-I(gamma)	PTU-I1
p2019C-3201_2	CP090858	61,933	560	IncFII(pHN7A8), IncFII(pSFO)	PTU-F_E_
p2019C-3201_3	CP090859	92,724	623	IncFIB, IncFII	PTU-E5

*PTU, plasmid taxonomic unit.

We selected a subset of 245 isolates for further genomic analysis to more evenly sample across outbreaks and to reduce computational demands. Isolates were characterized by core genome MLST implemented in BioNumerics 7.6 (*6*) and high-quality single-nucleotide polymorphism (SNP; hqSNP) methods using Lyve-SET version 1.1.4f (*9*), using the chromosomal sequence of 2019C-3201 as a reference and the Lyve-SET presets for *E. coli*. Overall, hqSNP was more discriminatory, differentiating isolates by a median of 10 pairwise hqSNPs (0–39 SNPs), whereas cgMLST differentiated isolates by a median of 2 allele differences (0–8 alleles) (Table 1). This finding was foreseeable because hqSNP does not depend on a predefined scheme; therefore, intergenic SNPs between loci, multiple SNP differences within a given locus, or SNPs in loci not included in the cgMLST schema can result (*9*).

**Figure 1:**
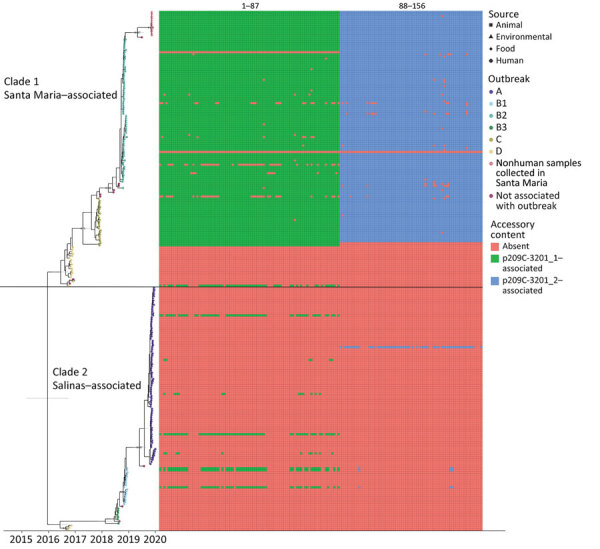
Tip-dated maximum clade credibility tree of 245 isolates of reoccurring *Escherichia coli* O157:H7 strain REPEXH02 linked to leafy greens–associated outbreaks, 2016–2019, generated in BEAST2 (https://www.beast2.org). Tips are aligned with the date of collection; calendar year is shown on the x-axis. Tips are colored according to the outbreak to which each isolate belonged; the shape corresponds to sample type (e.g., human, animal, environmental, or food). A horizontal black line segregates the two identified clades. Clade 1 contains outbreak B2 where some illness was traced back to Santa Maria, California, USA, as well as environmental samples collected in that region. Clade 2 contains outbreak A, which was traced back to the Salinas Valley, California. The presence/absence matrix to the right of the tree displays accessory genome content identified using Roary/scoary with 90% sensitivity and specificity to a subset of clade 1 isolates. A legend for accessory genome feature labels is included in Appendix 1 Table 5.

Time-tree analysis using BEAST version 2.6.3 (*10*) revealed the divergence of this strain into 2 clades that last shared a common ancestor around late 2015 (median December 19, 2015; 95% highest posterior density interval December 7, 2014–July 10, 2016) (Figure 1). After outbreak D in 2016, sequences corresponding to a given outbreak belonged to 1 of 2 clades; outbreaks B2 and C were associated with clade 1, and outbreaks A, B1, and B3 were associated with clade 2. Of note, outbreak A was traced to romaine lettuce from Salinas Valley, whereas traceback and sampling in outbreak B2 linked some illnesses to romaine lettuce from Santa Maria, California (https://www.fda.gov/food/outbreaks-foodborne-illness/investigation-summary-factors-potentially-contributing-contamination-romaine-lettuce-implicated-fall; https://www.fda.gov/food/outbreaks-foodborne-illness/outbreak-investigation-e-coli-romaine-salinas-california-november-2019). Lettuce from Salinas was not considered a source of any illnesses in outbreak B2. Environmental sampling in Santa Maria in 2019 yielded isolates clustering closely with outbreak B2 in the time tree.

We analyzed the closed reference sequence of 2019C-3201 using Prokka version 1.8 to enable SNP annotation (*11*). We examined output from Lyve-SET to determine the SNPs differentiating the 2 clades in our phylogenetic analysis (Appendix 1 Table 2). This work confirms a previous study reporting a nonsense mutation in the *arsR* gene, an arsenical resistance operon repressor (*12*). All clade 1 isolates in this study possess a G→A mutation resulting in a premature stop codon. This mutation could decrease the activity of this repressor and lead to constitutive expression of this operon. Agricultural soils and water sources can contain increased arsenic levels because of natural processes, industrial sources, or agricultural uses of arsenic, such as application of arsenic-containing herbicides, pesticides, or animal drugs (*13*). This mutation could provide an ecologic advantage in environments containing high levels of arsenic. This finding underscores the potential need to routinely screen enteric bacterial strains for heavy metal resistance determinants, as well as to consider heavy metal levels in soil as part of traceback investigations.

**Figure 2:**
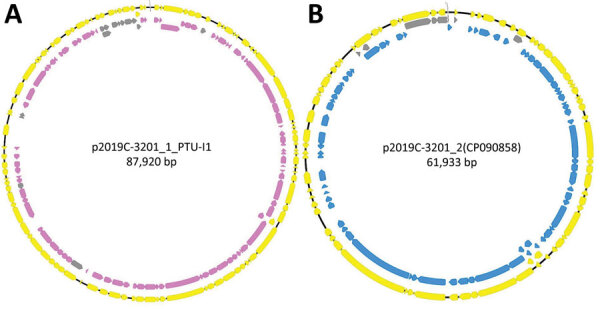
Annotated plasmids of reference genome 2019C-3201 of *Escherichia coli* O157:H7 containing clade-specific genomic features. A) p2019C-3201_1 annotated with prokka version 1.14.5 (yellow annotations) (*11*). Mapped list of Roary features (pan_genome_references.fa) onto plasmid (95% nucleotide identity; gray annotations) (*14*). Features highlighted had >90 sensitivity and >90 specificity to a subset of clade 1 isolates (pink annotations). The region with specific/sensitive features covers a large portion of the plasmid and predominately contains genes encoding hypothetical proteins with unknown functions and common plasmid-associated genes. Three features did not map in Geneious because they were either below 95% identity (2 features) or were identified as partial copy (1 feature). B) p2019C-3201_2 annotated with prokka v1.14.5 (yellow annotations). Mapped list of Roary features (pan_genome_references.fa) onto plasmid (100% nucleotide identity; gray annotations). Features highlighted had >90 sensitivity and >97 specificity (blue annotations) to a subset of clade 1 isolates. The region with specific/sensitive features covers a large portion of the plasmid and is associated with conjugation. Image was generated using Geneious version 2021.2 (https://www.geneious.com).

We further characterized isolates through assembly and annotation using Shovill-SPAdes version 1.0.9 and Prokka version 1.14.5 (*11*) and subsequent analysis in Roary version 3.11.2 (*14*) and scoary version 1.6.16 (*15*) to identify differences in the pangenome among isolates. We compared differentially distributed genes with the reference genome using BLASTn (https://blast.ncbi.nlm.nih.gov/Blast.cgi) to identify feature location (chromosome/plasmid). Roary/scoary analysis revealed a subset of clade 1 isolates with additional genomic content. A total of 156 genomic features had >90 sensitivity and >90 specificity to this subset of clade 1. Of those, 87 (56%) are on plasmid p2019C-3201_1, and 69 (44%) are on p2019C-3201_2 (Figure 2; Appendix 1 Tables 3, 4). Prokka-annotated features associated with p2019C-3201_1 (Figure 2; Appendix 1 Table 3) were predominantly genes encoding hypothetical proteins with unknown functions and common plasmid-associated genes. Annotated features associated with p2019C-3201_2 (Figure 2; Appendix 1 Table 4) were predominantly associated with conjugation and span a large portion of that plasmid. Additional work is necessary to characterize the role of these plasmids in clade 1. When visualizing the distribution of these clade 1–specific features alongside the maximum-clade credibility tree (Figure 1; Appendix 1 Table 5), it appears those features were acquired after clade 1 and clade 2 diverged. Given the geographic distribution of isolates, these features might be a result of adaptation to a particular niche or environment.

## Conclusions

In summary, a specific strain of *E. coli* O157:H7 associated with leafy greens has been the source of ongoing enteric illness since late 2016. This strain is estimated to have emerged in late 2015 and consists of 2 clades with different geographic distributions, 1 of which has notable genomic features. After this analysis, an additional outbreak associated with this strain was detected in late 2020 in which a reported 40 infections occurred in 19 states; 20 persons were hospitalized, and 4 developed hemolytic uremic syndrome (https://www.cdc.gov/ecoli/2020/o157h7-10-20b/index.html). After that outbreak, no further outbreaks have been detected, and only a single clinical isolate associated with this strain has been identified by PulseNet. The Centers for Disease Control and Prevention has classified this strain as a reoccurring, emerging, or persistent (REP) strain (https://www.cdc.gov/ncezid/dfwed/outbreak-response/rep-strains.html) with the designation REPEXH02. REP strains represent a new paradigm in enteric molecular surveillance, distinct from discrete outbreaks where numerous cases occur in a relatively short time frame. Detailed genomic characterization of additional REP strains, using the types of approaches outlined in this study, is necessary to elucidate factors contributing to their emergence and persistence in specific environments.

Appendix 1 Additional data used in study of reoccurring *Escherichia coli* O157:H7 strain REPEXH02 linked to leafy greens–associated outbreaks, 2016–2019

Appendix 2 Additional information about reoccurring *Escherichia coli* O157:H7 strain REPEXH02 linked to leafy greens–associated outbreaks, 2016–2019

## References

[1] Scallan E, Hoekstra RM, Angulo FJ, Tauxe RV, Widdowson MA, Roy SL, et al. Foodborne illness acquired in the United States—major pathogens. Emerg Infect Dis. 2011;17:7–15. 10.3201/eid1701.P11101 21192848 PMC3375761

[2] Mead PS, Griffin PM. *Escherichia coli* O157:H7. Lancet. 1998;352:1207–12. 10.1016/S0140-6736(98)01267-7 9777854

[3] Bielaszewska M, Schmidt H, Liesegang A, Prager R, Rabsch W, Tschäpe H, et al. Cattle can be a reservoir of sorbitol-fermenting shiga toxin-producing *Escherichia coli* O157:H(-) strains and a source of human diseases. J Clin Microbiol. 2000;38:3470–3. 10.1128/JCM.38.9.3470-3473.2000 10970407 PMC87410

[4] Heiman KE, Mody RK, Johnson SD, Griffin PM, Gould LH. *Escherichia coli* O157 Outbreaks in the United States, 2003-2012. Emerg Infect Dis. 2015;21:1293–301. 10.3201/eid2108.141364 26197993 PMC4517704

[5] Marshall KE, Hexemer A, Seelman SL, Fatica MK, Blessington T, Hajmeer M, et al. Lessons learned from a decade of investigations of Shiga toxin–producing *Escherichia coli* outbreaks linked to leafy greens, United States and Canada. Emerg Infect Dis. 2020;26:2319–28. 10.3201/eid2610.191418 32946367 PMC7510726

[6] Tolar B, Joseph LA, Schroeder MN, Stroika S, Ribot EM, Hise KB, et al. An overview of PulseNet USA databases. Foodborne Pathog Dis. 2019;16:457–62. 10.1089/fpd.2019.2637 31066584 PMC6653802

[7] Lin Y, Yuan J, Kolmogorov M, Shen MW, Chaisson M, Pevzner PA. Assembly of long error-prone reads using de Bruijn graphs. Proc Natl Acad Sci U S A. 2016;113:E8396–405. 10.1073/pnas.1604560113 27956617 PMC5206522

[8] Redondo-Salvo S, Bartomeus-Peñalver R, Vielva L, Tagg KA, Webb HE, Fernández-López, et al. COPLA, a taxonomic classifier of plasmids. BMC Bioinfo. 2021;22:390. 10.1186/s12859-021-04299-x PMC8325299 34332528

[9] Katz LS, Griswold T, Williams-Newkirk AJ, Wagner D, Petkau A, Sieffert C, et al. A comparative analysis of the Lyve-SET phylogenomics pipeline for genomic epidemiology of foodborne pathogens. Front Microbiol. 2017;8:375. 10.3389/fmicb.2017.00375 28348549 PMC5346554

[10] Bouckaert R, Vaughan TG, Barido-Sottani J, Duchêne S, Fourment M, Gavryushkina A, et al. BEAST 2.5: An advanced software platform for Bayesian evolutionary analysis. PLOS Comput Biol. 2019;15:e1006650. 10.1371/journal.pcbi.1006650 30958812 PMC6472827

[11] Seemann T. Prokka: rapid prokaryotic genome annotation. Bioinformatics. 2014;30:2068–9. 10.1093/bioinformatics/btu153 24642063

[12] Cherry JL. Recent genetic changes affecting enterohemorrhagic *Escherichia coli* causing recurrent outbreaks. Microbiol Spectr. 2022;10:e0050122. 10.1128/spectrum.00501-22 35467376 PMC9241674

[13] Punshon T, Jackson BP, Meharg AA, Warczack T, Scheckel K, Guerinot ML. Understanding arsenic dynamics in agronomic systems to predict and prevent uptake by crop plants. Sci Total Environ. 2017;581-582:209–20. 10.1016/j.scitotenv.2016.12.111 28043702 PMC5303541

[14] Page AJ, Cummins CA, Hunt M, Wong VK, Reuter S, Holden MTG, et al. Roary: rapid large-scale prokaryote pan genome analysis. Bioinformatics. 2015;31:3691–3. 10.1093/bioinformatics/btv421 26198102 PMC4817141

[15] Brynildsrud O, Bohlin J, Scheffer L, Eldholm V. Rapid scoring of genes in microbial pan-genome-wide association studies with Scoary. Genome Biol. 2016;17:238. 10.1186/s13059-016-1108-8 27887642 PMC5124306

