# Supplementary material for: Reoccurring Escherichia coli O157:H7 Strain Linked to Leafy Greens–Associated Outbreaks, 2016–2019
Source: Emerg Infect Dis. 2023 Sep;29(9):1895–9. doi: 10.3201/eid2909.230069 (PMC10461648; doi:10.3201/eid2909.230069)
Supplement: Appendix 2 — Additional information about reoccurring Escherichia coli O157:H7 strain REPEXH02 linked to leafy greens–associated outbreaks, 2016–2019 [file 23-0069-Techapp-s2.pdf]

Article DOI: https://doi.org/10.3201/eid2909.230069

EID cannot ensure accessibility for supplementary materials supplied by authors. Readers who have difficulty accessing supplementary content should contact the authors for assistance.

Reoccurring *Escherichia coli* O157:H7 Strain Linked to Leafy Greens–Associated Outbreaks, 2016–2019

Appendix 2

Supplemental Methods

Reference genome generation and characterization

We generated a closed reference genome, 2019C-3201 (WGSID: PNUSAE020169), using PacBio Sequel technology and assembled with Flye v.2.6 with the –plasmids option (*1*). The final assembly was then polished with Illumina reads using unicycler_polish scripts from Unicycler suite version v0.4.8 (*2*). Plasmids were characterized using a modified PlasmidFinder database (https://github.com/StaPH-B/resistanceDetectionCDC/blob/master/plasmidDatabase.fasta) (*3*) and COPLA (*4*) to identify plasmid replicons and plasmid taxonomic units respectively.

Genomic subtyping

MLST was determined using *mlst* (https://github.com/tseemann/mlst) with the Achtman seven-gene scheme. Stx gene subtype(s) were determined using in-silico PCR (https://github.com/ucscGenomeBrowser/kent/tree/master/src/isPcr) with the primers described by Flemming, et al. (*5*). The O157 Manning Clade was determined by identifying the four informative SNPs at loci ECs2357, ECs2521, ECs3881, and ECs4130. The matching clade was assigned based on these SNPs as described by Riordan, et al (*6*).

Phylogenetic analysis

Isolates were characterized by high-quality single nucleotide polymorphism (hqSNP) methods using Lyve-SET v.1.1.4f (*7*), using the chromosomal sequence of 2019C-3201 as a

reference and the Lyve-SET presets for *Escherichia coli*. The closed reference sequence of 2019C-3201 was analyzed using Prokka v1.8 to facilitate SNP annotation (*8*). To generate a time tree in BEAST v.2.6.3 (*9*) the alignment from Lyve-SET was processed using gubbins 3.0.0 (*10*) to generate a recombination-free SNP alignment. The resulting tree was first analyzed in TempEST v.1.5.3 (*11*) to assess suitability for molecular clock analysis. The resulting alignment was analyzed in BEAST2 while accounting for constant sites by modifying the xml file from Beauti to include <data id = ”filt” spec = ”FilteredAlignment” filter = ”-” data = ”@filtOriginal” constantSiteWeights = ”470006 589755 590021 468034”/>. bModelTest v1.2.1 (*12*) was employed to average across appropriate substitution models. All coalescent tree priors under both a strict and relaxed clock and were evaluated with an initial clock rate of 5.0x10^{-7} SNPs/site/year, and analyses were run for 500,000,000 iterations with sampling every 50,000 iterations. A maximum clade credibility tree was generated using TreeAnnotator and the median options for height. The resulting time tree was visualized using the ggtree package (*13*) for R v.4.1.1.

Pangenome analysis

Isolates were assembled and annotation using Shovill-spades v.1.0.9 (https://github.com/tseemann/shovill; with the options–trim,–depth 100,–mincov set to 10% average genome coverage) and Prokka v.1.14.5 (*8*). Subsequent analysis in Roary v.3.11.2 (*14*) and scoary v.1.6.16 (*15*) using default parameters was performed to identify differences in the pangenome among isolates.

References

1. Lin Y, Yuan J, Kolmogorov M, Shen MW, Chaisson M, Pevzner PA. Assembly of long error-prone reads using de Bruijn graphs. Proc Natl Acad Sci U S A. 2016;113:E8396–405. PubMed https://doi.org/10.1073/pnas.1604560113

2. Wick RR, Judd LM, Gorrie CL, Holt KE. Unicycler: resolving bacterial genome assemblies from short and long sequencing reads. PLOS Comput Biol. 2017;13:e1005595. PubMed https://doi.org/10.1371/journal.pcbi.1005595

3. Carattoli A, Zankari E, García-Fernández A, Voldby Larsen M, Lund O, Villa L, et al. In silico detection and typing of plasmids using PlasmidFinder and plasmid multilocus sequence typing.

Antimicrob Agents Chemother. 2014;58:3895–903. PubMed https://doi.org/10.1128/AAC.02412-14

4) Redondo-Salvo S, Bartomeus-Peñalver R, Vielva L, Tagg KA, Webb HE, Fernández-López, et al. COPLA, a taxonomic classifier of plasmids. BMC Bioinformatics. 2021; 22:390. **PMID 34332528**

5. Scheutz F, Teel LD, Beutin L, Piérard D, Buvens G, Karch H, et al. Multicenter evaluation of a sequence-based protocol for subtyping Shiga toxins and standardizing Stx nomenclature. J Clin Microbiol. 2012;50:2951–63. PubMed https://doi.org/10.1128/JCM.00860-12

6. Riordan JT, Viswanath SB, Manning SD, Whittam TS. Genetic differentiation of *Escherichia coli* O157:H7 clades associated with human disease by real-time PCR. J Clin Microbiol. 2008;46:2070–3. PubMed https://doi.org/10.1128/JCM.00203-08

7. Katz LS, Griswold T, Williams-Newkirk AJ, Wagner D, Petkau A, Sieffert C, et al. A comparative analysis of the Lyve-SET phylogenomics pipeline for genomic epidemiology of foodborne pathogens. Front Microbiol. 2017;8:375 PubMed https://doi.org/10.3389/fmicb.2017.00375

8. Seemann T. Prokka: rapid prokaryotic genome annotation. Bioinformatics. 2014;30:2068–9. PubMed https://doi.org/10.1093/bioinformatics/btu153

9. Bouckaert R, Vaughan TG, Barido-Sottani J, Duchêne S, Fourment M, Gavryushkina A, et al. BEAST 2.5: an advanced software platform for Bayesian evolutionary analysis. PLOS Comput Biol. 2019;15:e1006650. PubMed https://doi.org/10.1371/journal.pcbi.1006650

10. Croucher NJ, Page AJ, Connor TR, Delaney AJ, Keane JA, Bentley SD, et al. Rapid phylogenetic analysis of large samples of recombinant bacterial whole genome sequences using Gubbins. Nucleic Acids Res. 2015;43:e15. PubMed https://doi.org/10.1093/nar/gku1196

11. Rambaut A, Lam TT, Max Carvalho L, Pybus OG. Exploring the temporal structure of heterochronous sequences using TempEst (formerly Path-O-Gen). Virus Evol. 2016;2:vew007. PubMed https://doi.org/10.1093/ve/vew007

12. Bouckaert RR, Drummond AJ. bModelTest: Bayesian phylogenetic site model averaging and model comparison. BMC Evol Biol. 2017;17:42. PubMed https://doi.org/10.1186/s12862-017-0890-6

13. Yu G, Smith D, Zhu H, Guan Y, Lam TT. ggtree: an R package for visualization and annotation of phylogenetic trees with their covariates and other associated data. Methods Ecol Evol. 2017;8:28–36. https://doi.org/10.1111/2041-210X.12628

14. Page AJ, Cummins CA, Hunt M, Wong VK, Reuter S, Holden MTG, et al. Roary: rapid large-scale prokaryote pan genome analysis. Bioinformatics. 2015;31:3691–3. PubMed https://doi.org/10.1093/bioinformatics/btv421

15. Brynildsrud O, Bohlin J, Scheffer L, Eldholm V. Rapid scoring of genes in microbial pan-genome-wide association studies with Scoary. Genome Biol. 2016;17:238. https://doi.org/10.1186/s13059-016-1108-8

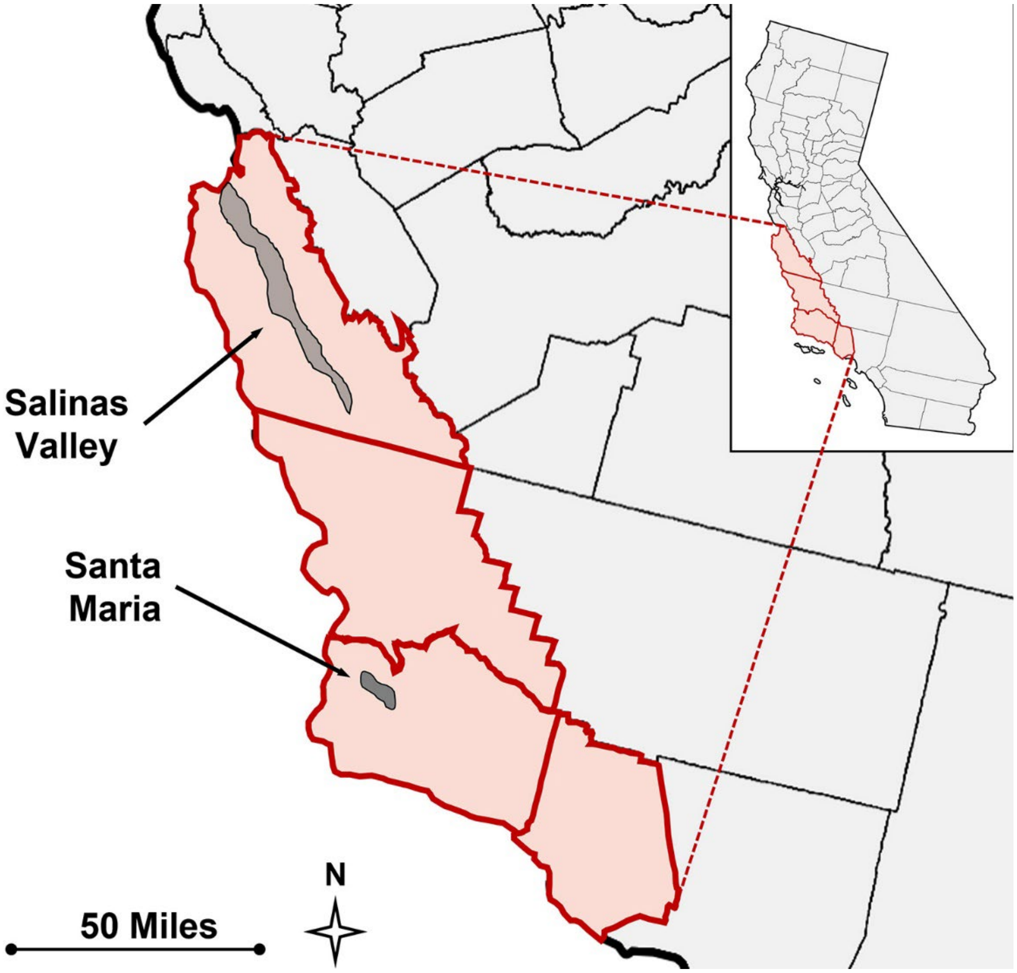

Appendix 2 Figure. Map illustrating growing regions in California, USA, of which some REPEHX02-linked illnesses are associated with. More specifically, outbreak A in this study was traced to romaine lettuce from Salinas Valley, California, while traceback and sampling in outbreak B2 linked some illnesses to romaine lettuce from Santa Maria, California, both labeled and shaded in grey on the map. Image adapted with permission from the Food and Drug Administration (Original source: https://www.fda.gov/media/147349/download#:~:text=As%20a%20result%20of%20this,and%202020%20leafy%20greens%20outbreaks.
